# Cadence discovery: study protocol for a dose-finding and mechanism of action clinical trial of sodium benzoate in people with treatment-refractory schizophrenia

**DOI:** 10.1186/s13063-021-05890-6

**Published:** 2021-12-13

**Authors:** Andrea Baker, Lachlan Clarke, Peter Donovan, Jacobus P. J. Ungerer, Gunter Hartel, George Bruxner, Luca Cocchi, Anne Gordon, Vikas Moudgil, Gail Robinson, Digant Roy, Ravinder Sohal, Emma Whittle, James G. Scott

**Affiliations:** 1 QIMR Berghofer Medical Research Institute, Herston, QLD Australia; 2 Queensland Centre for Mental Health Research, The Park Centre for Mental Health, Wacol, QLD Australia; 3 Faculty of Medicine, The University of Queensland, Herston, QLD Australia; 4 Clinical Pharmacology, Royal Brisbane and Women’s Hospital, Herston, QLD 4006 Australia; 5 School of Biomedical Sciences, The University of Queensland, Herston, QLD Australia; 6 Pathology Queensland, Royal Brisbane and Women’s Hospital, Herston, QLD Australia; 7 Metro North Mental Health Service, Caboolture Hospital, Caboolture, QLD Australia; 8 Metro North Mental Health Service, The Prince Charles Hospital, Chermside, QLD Australia; 9 Metro North Mental Health Service, Royal Brisbane and Women’s Hospital, Herston, QLD Australia

**Keywords:** Schizophrenia, Adjunctive, Treatment refractory, Sodium benzoate, Intervention, RCT, Clinical trial, PANSS

## Abstract

**Background:**

Schizophrenia is a persistent psychotic disorder often accompanied by severe disability and premature mortality. New pharmacological treatments are urgently needed. Sodium benzoate, a common food preservative holds potential to be an effective, accessible treatment for schizophrenia, though the optimal dosing and mechanism of action of the compound requires further investigation.

**Methods:**

Individuals with persistent treatment-refractory schizophrenia (*n*=52) will be recruited. Patients will be randomised in a 1:1:1:1 ratio to receive treatment of one of three active doses (1000, 2000 or 4000 mg daily) of sodium benzoate or placebo for 6 weeks duration. The primary outcome measurement is change in the Positive and Negative Syndrome Scale (PANSS) total score. Secondary outcome measurements are PANSS subscales, Global Assessment of Function (GAF), Clinical Global Impression (CGI) and Patient Global Impression (PGI-I). Change in concentrations of peripheral amino acids (D-alanine, L-alanine, D-serine, L-serine, glycine and glutamate), plasma sodium benzoate, plasma catalase, 3-nitrotyrosine, malondialdehyde and high-sensitivity C-reactive protein (hs-CRP) will be determined as tertiary measures.

**Discussion:**

This trial seeks to build upon previous research indicating potential efficacy of sodium benzoate for reduction of symptoms in individuals with treatment-refractory schizophrenia. The trial aims to improve the understanding of the mechanism of action of the compound.

**Trial registration:**

Australian New Zealand Clinical Trials Registry (ANZCTR) ACTRN12621000327886. Registered on 23 March 2021.

## Background

Schizophrenia is a persistent psychotic disorder all too often accompanied by lifelong adverse impact on many facets of life for affected individuals and their families. The symptoms of the syndrome are heterogeneous, frequently categorised into positive, negative and cognitive domains [1]. There is a significant burden of disease attributable to schizophrenia due to its onset commonly occurring in adolescence and young adulthood combined with its persistence, high level of associated disability [2] and excess mortality [3, 4].

### Current therapeutic interventions

Antipsychotics acting through modulation of the dopamine receptor are the most commonly used medicinal treatment for schizophrenia, albeit with high rates of side effects and modest clinical efficacy which often leads to discontinuation [1, 5, 6]. Treatment-refractory schizophrenia is the term used when the illness does not respond to antipsychotic therapy [7]. Although there are no definitive data on the incidence/prevalence of treatment-refractory schizophrenia, a cohort study in the UK (*n*=246) observed that 33% of patients could be classified as treatment-resistant within 5 years of first episode psychosis, and 23% never achieved symptomatic remission [8]. General estimates suggest that up to 40% of people with schizophrenia are treatment-refractory [6]. Clozapine is the recommended medication for those with treatment-refractory schizophrenia, although less than half of people respond and there are significant adverse effects including commonly occurring metabolic syndrome and sedation and uncommon but severe haematological and cardiac side effects [9, 10]. Thus, finding effective and tolerable therapeutics for treatment-resistant schizophrenia is a clinical priority.

### Alternative therapeutic target: the NMDA receptor

The need for novel pharmacological agents has stimulated interest in developing therapeutics which modulate the N-methyl-D-aspartate (NMDA) receptor. Hypofunction of the NMDA receptor has been implicated in the pathophysiology of schizophrenia through clinical, genetic and molecular studies [11–15]. The NMDA receptor consists of 2 subunits, glycine and glutamate. Modulation of the glutamate subunit is limited due to excitotoxicity resulting from elevated glutamate levels [16]. Activation of the NMDA receptor by the amino acids glycine and D-serine results in an improvement in all symptom domains of schizophrenia [17]. However, the use of compounds such as D-serine for treating schizophrenia is inhibited by its limited availability in the central nervous system due to its metabolism by the flavoenzyme D-amino-acid oxidase (DAAO) [18]. Further, there are concerns that the metabolites arising from high doses of D-serine in humans may confer nephrotoxicity [19, 20]. Adding an inhibitor of DAAO with D-serine has been observed to increase the levels of D-serine in the plasma and central nervous system relative to D-serine administration alone in rats [21]. A potent inhibitor of DAAO is sodium benzoate, a commonly used food preservative and metabolite of cinnamon. To date there have been four published clinical trials using the compound for treatment of psychosis [22–25].

### Evidence for the effectiveness of sodium benzoate in psychotic illnesses

Lane et al. [22] published the first randomised clinical trial (*n*=52) of sodium benzoate for patients with chronic, stable schizophrenia, examining the effectiveness of 1000 mg/day adjunct sodium benzoate medication over 6 weeks. Those receiving sodium benzoate had greater improvements as measured by changes in the Positive and Negative Syndrome Scale (PANSS) total, PANSS positive, PANSS negative and PANSS general psychopathology with large effect sizes [22]. A second trial followed where 63 people with chronic schizophrenia were randomised to receive either combined sarcosine (2000 mg/day) and sodium benzoate (1000 mg/day), sarcosine only or placebo in addition to their usual antipsychotic therapy for 12 weeks. Those who received combined sarcosine and sodium benzoate had improvements in cognition and global functioning compared with those in the other groups [25]. A third trial of participants with treatment-refractory schizophrenia (*n*=60) examined the effect of 1000 mg and 2000 mg daily adjunct sodium benzoate for patients stabilised with clozapine over 6 weeks [23]. Both 1000 mg and 2000 mg groups significantly outperformed placebo in the Scale for the Assessment of Negative Symptoms (SANS) scores whilst only the 2000 mg group did better than those given placebo in total PANSS, Quality of Life Scale (QOLS) and Positive PANSS subscale [23]. This indicates a potential dose-dependent increase in efficacy of sodium benzoate. Finally, an RCT comparing adjunctive sodium benzoate (1000 mg/day) with placebo over 12 weeks for individuals with early psychosis (*n*=100) [26], most of whom had early schizophrenia (*n*=83) showed no significant difference in the change in PANSS scores between the two groups [24]. This lack of improvement from sodium benzoate relative to placebo may have been due to participants in both the intervention and control arms improving substantially over the duration of the trial because of the early illness which was treatment responsive. Alternatively, it may have been a result of insufficient sodium benzoate dose or another biological mechanism causing sodium benzoate to be more efficacious in people with chronic schizophrenia rather than early illness.

### Need for a dose-finding and mechanism of action trial

Uncertainty remains regarding the optimal sodium benzoate dose for the treatment of people with schizophrenia. The original dose-finding research was an open-label pilot study conducted on five participants where three received doses of 1000 mg/day and had reductions in PANSS scores. The other two received lower doses and showed no improvement [22]. This research informed the original trials that used 1000 mg/day [22, 25]. A subsequent trial reported that 2000 mg/day was more effective at reducing symptoms than 1000 mg/day, though it is not known whether further improvement can be achieved by doses greater than 2000 mg/day [23].

Likewise, uncertainty also exists regarding the mechanism of action through which sodium benzoate acts in psychotic illnesses [27]. The original hypothesis was that sodium benzoate increases the bioavailability of D-amino acids by inhibiting DAAO, which modulated the activity of the glutamate sub-unit of the NMDA receptors [22]. However, two recent trials have reported no changes in peripheral D-amino acid concentration following sodium benzoate administration [23, 24]. This could be the result of peripheral D-amino acid concentration not being reflective of brain D-amino acid concentration or that the dose of sodium benzoate was insufficiently high to achieve significant change. However, administration of sodium benzoate to mice who were injected with the NMDA receptor antagonist phencyclidine (PCP), attenuated the observed motor effects of PCP but did not result in changes in plasma or brain concentrations of D-serine [28]. Alternatively, reduction in oxidative stress has been  proposed as a potential alternate mechanism of sodium benzoate action [29], as oxidative stress has been associated with schizophrenia [30]. Lin et al. [23] observed an increase in peripheral catalase concentration of the 2000 mg/day sodium benzoate treated group relative to placebo and reported that catalase concentration was significantly negatively correlated with PANSS total and PANSS positive scores, congruent with evidence that red blood cell catalase is a state marker for schizophrenia [30]. Thus, we intend to examine the efficacy of sodium benzoate at three dose levels with the aim to clarify the efficacy and as well as to further examine the mechanism of action of sodium benzoate safety at the three doses.

### Safety profile of sodium benzoate

The safety of sodium benzoate has been studied extensively because it has been widely used as a food preservative, in cosmetics and also as a medication. The US Food and Drug Administration has classified sodium benzoate as ‘Generally Recognized As Safe’ and regulates the concentration of sodium benzoate to 0.1% by weight in food products and 1% concentration in medicines [31, 32]. The joint committee by the Food and Agriculture Organization of the United Nations and the World Health Organization has suggested an acceptable daily intake of 0.5 mg/kg of body weight [33].

The International Programme on Chemical Safety (IPCS) published a report on sodium benzoate (and a related compound benzoic acid) detailing the potential health effects of sodium benzoate in animal studies. Testing in rodents revealed a low rate of toxicity with mean lethal dose (LD50) values of > 1940 mg/kg body weight. Drawing evidence from two long term studies (12–16 months) in rodents, there was no evidence to suggest sodium benzoate had carcinogenic properties. Likewise, studies of the precursors of benzoic acid–benzyl acetate, benzyl alcohol, and benzaldehyde suggest that a carcinogenic effect of sodium benzoate is unlikely. The results of genotoxic activity were inconclusive in the IPCS report, and there were no consistent abnormal findings based on the Ames test. Based on in vitro studies of human lymphoblastoid cell lines, the evidence suggests that sodium benzoate at very high concentrations does have genotoxic effects. Sodium benzoate does have embryotoxic and fetotoxic effects; however, these are only evident at dosage levels high enough to cause severe maternal toxicity. A No Observable Adverse Effect Level (NOAEL) of approximately 1310 mg/kg body weight for teratogenic effects in rodents was established [34].

The acute toxicity of oral sodium benzoate in humans is low. There is evidence that some atopic individuals may be sensitive to food additives and preservatives (benzoate is a food preservative) [35]. The safety of sodium benzoate  reported in three trials of people with psychosisshowed no increase in reported side effects in participants who received sodium benzoate compared to placebo [22–24].

Sodium benzoate has been used since the late 1970s to treat patients with urea cycle enzymopathies that cause hyperammonaemia [36, 37]. The therapeutic dose administered to treat hyperammonaemia over several years is in the range of 250–500 mg/kg body weight per day, which equates to 17,500–35,000 mg per day for a body weight of 70 kg. It is noted that at this dose level, the clinical signs of toxicity are rare and are limited to anorexia and vomiting, especially after large intravenous bolus injections with 100% bioavailability.

### Objectives and hypothesis

The primary objective of the study is to determine the impact of 1000 mg daily, 2000 mg daily and 4000 mg daily of sodium benzoate treatment over 6 weeks on the total PANSS scores in patients with treatment-refractory schizophrenia compared to placebo.

The secondary objectives of the study are to determine the impact of (1000 mg daily or 2000 mg daily or 4000 mg daily) of sodium benzoate treatment over 6 weeks on the Positive and Negative Syndrome Scale (PANSS) subscales, Global Assessment of Function (GAF), Clinical Global Impression (CGI) and Patient Global Impression of Improvement (PGI-I), compared to individuals taking placebo. A further secondary objective is to determine the safety and tolerability of the three doses of sodium benzoate under investigation.

A tertiary (exploratory) objective is to determine the mechanism of action by examining the change in biochemical markers relative to baseline and correlate these with clinical outcomes. These biochemical markers consist of plasma amino acids (D-alanine, L-alanine, D-serine, glycine, glutamine and glutamate), plasma concentration of sodium benzoate, plasma catalase, 3-nitrotyrosine, malondialdehyde and high sensitivity CRP (hs-CRP).

It is hypothesised that participants allocated to the active arms (1000, 2000 or 4000 mg daily) will have a significant reduction in PANSS total score compared to the participants allocated placebo. This reduction is also expected in the secondary scores. It is also hypothesised that in the event of successful treatment with sodium benzoate, biochemical changes associated with the proposed mechanism of action will be observed, notably an increase in D-amino acids (D- and L-serine, D- and L-alanine, D- and L-glutamate) and decreased oxidative stress.

### Study design

The design is a randomised, placebo-controlled, double-blind parallel-group trial to determine the dose of sodium benzoate required for add-on treatment of patients with treatment-refractory schizophrenia. The study is a phase II dose-finding study with secondary endpoints which comprise a pharmacokinetic/pharmacodynamics investigation. This study will include 52 individuals with treatment-refractory schizophrenia. Patients will be given 1000 mg (500 mg bd), 2000 mg (1000 mg bd) or 4000 mg (2000 mg bd) daily of sodium benzoate or placebo. Participants will receive either active treatment (1000, 2000 or 4000 mg/day) or placebo in a 1:1:1:1 ratio. This study medication will be in addition to participants’ routine care, defined as ‘individualized combinations of psychopharmacology, behavioural interventions, rehabilitation and associated clinical services’ in keeping with Queensland Health standards of care. Face-to-face clinical assessments will be at baseline (week 0) and weeks 2, 4 and 6. Weekly phone contact will occur in weeks between face-to-face visits. The SPIRIT reporting guidelines have been used in this study protocol and a spirit checklist has been completed [38].

### Study population

Fifty-two (52) participants will be recruited through the mental health services at Metro North Hospital and Health Service and West Moreton Hospital and Health Services in Queensland, Australia. Recruitment to the study will be promoted by trial investigators working as clinicians in the study sites. This will ensure staff and consumers at the study sites are well informed about the study.

### Inclusion and exclusion criteria

Patients will be invited to participate in the study if they meet all of the following criteria:

1. Aged between 18 and 64 years (inclusive).
2. Fulfil the DSM-IV criteria for schizophrenia, based on the Diagnostic Interview for Psychosis.
3. Met diagnostic criteria for schizophrenia for at least 12 months duration.
4. PANSS total score ≥70.
5. Received antipsychotic medications for a period of at least one continuous month prior to assessment for the study and remained symptomatic despite the medications.
6. Agreed to participate, has capacity to consent and is able to follow the study instructions and procedures.

Patients will be excluded from the study if they meet any one of the following criteria:

1. Known allergies to sodium benzoate (E211) or any part of the formulation of the investigational product.
2. Suspected allergies or known adverse reactions to food preservatives in general.
3. Unable to understand or communicate in English.
4. For female participants, those currently pregnant, or planning to become pregnant or lactating during the study period or are sexually active and using ineffective contraception.
5. Inability to follow the study instructions and procedures.

### Sample size justification

The primary analysis will be a mixed effect repeated measures analysis comparing total PANSS scores across 4 time points (baseline, 2, 4, and 6 weeks) across 4 dose groups (placebo, 1000 mg, 2000 mg and 4000 mg per day). A recent study by Scott et al. [24, 26] observed a standard deviation for total PANSS score of around 15 for all subjects, and closer to 10 for cases with baseline PANSS scores > 75. The autoregressive (AR(1)) correlation between weeks was estimated at 0.815. Assuming a decrease in PANSS scores similar to this previous study, this study has at least 80% power to detect an effect size of 0.55 between treatments with a sample size of 52 participants, incorporating an allowance for 20% attrition.

### Randomisation, allocation concealment and blinding procedure

Participants will be randomised into one of the treatment groups using a computer-generated randomisation table once written consent has been obtained and the baseline assessments have been completed. Clinicians from participating mental health services will inform potential participants about the study. If interested and agreeing to be contacted, a member of the research team who is independent of the person’s clinical care will communicate further details about the study with the potential participant. If still wishing to participate, consent from participants will be obtained by the researcher. Their capacity to understand the risks and benefits of participation will be assessed on an ongoing basis in compliance with Australian National Health and Medical Research Council guidelines regarding ethical conduct in human research. Participants will receive either active treatment (1000, 2000 or 4000 mg/day) or placebo in a 1:1:1:1 ratio.

The randomisation will be double-blind. An independent biostatistician will generate the randomisation list which will be provided to the manufacturer and to the Princess Alexandra Hospital Pharmacist. All medication will be blinded to the study personnel (including data analysts), research pharmacist and the patient. Sodium benzoate and placebo capsules will be identical in terms of packaging, appearance, colour and taste. Treatment allocations will not be disclosed to the Investigator or any study personnel before the database is locked, unless in an emergency which requires unblinding.

### Unblinding

Only in the event of a medical emergency which the Investigator feels cannot be adequately managed without knowing the identity of the study medication, will the treatment code be unblinded for a particular participant. In such an event, this would be done by the Princess Alexandra Hospital Pharmacist via a 24-h number. All cases of emergency unblinding will be documented on a Serious Adverse Event Form and reported to the sponsor within 24 h. Unblinded participants will be withdrawn from the study.

### Drug administration

**Fig. 1:**
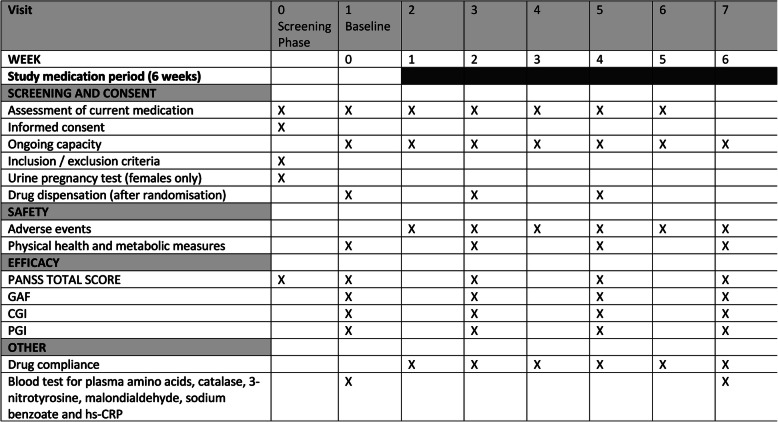
Schedule of visits and assessments. *PANSS* Positive and Negative Syndrome Scale, *GAF* Global Assessment of Functioning, *CGI* Clinical Global Impression, *PGI* Patients’ Global Impression of Improvement

The study medication will be compounded by Optima Ovest Pty Ltd. The medication for the active arms (1000, 2000 and 4000 mg/day) will consist of 4 doses of sodium benzoate (125 mg, 250 mg and 500 mg) in identical capsule form to be taken twice/day. The placebo will consist of microcrystalline cellulose in a matched gelatine capsule. Prior to dispensing, all study medication will be kept at the Queensland Centre for Mental Health Research, only accessible to delegated members of the study team. Dispensing to participants will occur once consent has been obtained and after the screening phase and randomisation has occurred. The study medication will be dispensed on a fortnightly basis by delegated research staff in accordance with Fig. 1*.* Compliance with study medication will be calculated at each visit by means of self-report and a capsule count. This data will be used to calculate compliance with medication for analysis purposes.

### Dose justification

With respect to the choice of doses of sodium benzoate to examine, Lane et al. [22] reported clinical efficacy with 1000 mg per day whilst Lin et al. [23] reported benefits of 2000 mg sodium benzoate per day that were larger than those from 1000 mg per day. No studies in people with schizophrenia have used doses of 4000 mg per day. Limited side effects are seen in the use of very high doses of up to 500 mg/kg/day in patients with urea cycle disorders [36] which gives us confidence to prescribe up to 4000 mg per day for the purposes of establishing the most appropriate dose for the treatment of schizophrenia.

### Comparator justification

The comparator condition in this study consists of placebo in addition to routine care. The Declaration of Helsinki affirms that placebo-controlled trials should only be used in the absence of existing proven therapy [39]. Therefore, the use of an adjunct therapy has been selected to ameliorate these ethical concerns as both the experimental and control groups will receive standard medical care (Treatment as Usual).

### Outcome measurement

Clinical measures of disease severity and physical health and biochemical samples will be taken at baseline and afterwards according to Fig. 1.

### Clinical outcome measures

The severity of schizophrenia symptoms as measured by the Positive and Negative Syndrome Scale (PANSS) total score will be the primary continuous outcome measure. The mean change in scores from baseline to week 6 (end of the study) will be compared between study arms.

Average change in scores from baseline to week 6 (end of the study) in the following secondary clinical outcome continuous measures will also be included:

- PANSS subscale scores, reflecting the severity of positive and negative symptoms and general psychopathology.
- Global Assessment of Function (GAF), a numeric scale (1 through 100) used by physicians to rate subjectively the social, occupational, and psychological functioning of adults.
- Clinical Global Impression (CGI) which is used to measure symptom severity, treatment response and the efficacy of treatments in treatment studies of patients with schizophrenia.
- Patient Global Impression (PGI) is a global index that is used to rate the response of a condition to a therapy (transition scale).

### Biomarkers

To investigate biochemical activity associated with sodium benzoate treatment a 25-mL sample of blood will be taken from consenting participants via venipuncture at baseline and week 6. Pathology Qld will be responsible for collecting, storing, and analysing blood samples. The biomarkers examined between study arms will include the average concentration of (1) plasma amino acids (D-alanine, L-alanine, D-serine, L-serine, glycine, glutamine and glutamate) and (2) plasma sodium benzoate. To assess for changes in oxidative stress, plasma catalase, 3-nitrotyrosine and malondialdehyde will be measured to determine whether sodium benzoate has a related mechanism of action, as suggested by a previous animal study [28] and human clinical trial [23]. It has been proposed that oxidative stress is involved in both the onset and the persistence of schizophrenia [30]. Average change in inflammatory response activity will be assessed by high-sensitivity CRP. Previous trials have not observed changes in D-amino acid concentration following sodium benzoate admission at 1000 mg and 2000 mg, inconsistent with the original hypothesised mechanism of action of the compound [23, 24]. Comparison of changes from baseline to week 6 (end of the study) in these biomarkers will allow us to observe whether administration of higher doses of sodium benzoate are needed to observe changes in peripheral amino acids. Participants refusing to for biological samples to be collected and stored can still participate in the trial.

### Compliance monitoring and adherence

After commencing the trial, participant’s adherence to medication will be assessed each week either through face-to-face visits or via phone. Participants will also be instructed to return any unused study medication and empty bottles to delegated research assistants. Assessment of compliance will be calculated by self-report and capsule count. Any identified issues with medication compliance will be discussed at weekly team meetings. To assist with participant compliance, a meals-reminder aid will be given with the study medication.

### Case report form

A case report form (CRF) will be completed for each study participant summarising all clinical screening and study data for analysis. In the CRF, participants will only be identified by their participant number in order to retain participant confidentiality.

The completed case report forms (CRF’s) will be retained by the Investigators for a period of at least 15 years or the maximum time frame as determined by local regulations, whichever is the longest.

### Study restrictions

There are no restrictions to participants during the study in terms of concomitant medication, exercise or ambulation.

### Safety assessments

All patients recruited in this study will be receiving care at either Metro North Hospital and Health Service or West Moreton Hospital and Health Service. The study team will liaise with clinical staff to ensure that participants have undergone a routine physical health screen. If a participant is of child-bearing potential and sexually active, urine pregnancy tests will be conducted at baseline and when clinically appropriate.

### Adverse events

The Investigator and designated study personnel will monitor each participant for adverse events during the study. All adverse events reported between consent and final follow-up visit will be recorded in the case report form (CRF). The investigator or designee will ask the participant non-leading questions in an effort to detect adverse events e.g. ‘Have you felt unwell or different in any way since your last visit’.

In addition, participants will be encouraged to spontaneously report any unusual feelings or sensations. Adverse events will be continuously monitored. Serious adverse event (death, life-threatening illness or incident, hospitalisation or any event deemed by the principal investigator to be a significant medical event) will be reported to the study sponsor, ethics committees and governance bodies and the DSMB within 24 h of notification of the incident. An assessment of causality from the investigational product for the serious adverse event will be completed at the time of notification.

### Participant withdrawal

All participants have the right to withdraw consent at any time without prejudice and this will not affect their ongoing care. This will be clearly discussed during the consenting process. If a participant decides to withdraw consent, we will complete a revocation of informed consent form.

Participants will be withdrawn from the study by the Investigator, prior to completion of treatment, under the following conditions:

- Non-compliant with study medication for seven consecutive days
- Non-adherence of more than 50% of study medication on capsule count
- Development of a serious adverse event assumed to be associated with the study medication
- Cessation of effective contraception or confirmed pregnancy
- Continual inability to provide informed consent.

### Early termination and stopping rules

As noted, the safety of the trial will be monitored on an ongoing basis throughout the study. The Data Safety Monitoring Board (DSMB) will conduct a formal evaluation of trial progress including considerations of participant risk vs. benefits after the recruitment of 25 participants or sooner if required (for instance, in the event of a serious adverse event). The study may be terminated prematurely by the Coordinating and/or Principal investigator or his/her designee, the DSMB, and the sponsor if:

- The number and/or severity of adverse events justify the discontinuation of the study.
- New data becomes available which raises concern about the safety of the study drug, so that continuation might cause unacceptable risks to participants.
- Recruitment is slow and it is assessed that the trial is unlikely to finish.

After such a decision, the Investigator or designee will contact all participants promptly, and written notification of study termination will be sent to the Reviewing Ethics Committee and relevant Governance Offices. A study closure advice will also be sent to the Therapeutic Goods Administration on the approved form. The Australian Clinical Trial Registry entry will also be updated accordingly.

### Participant reimbursement and compensation

Participants will be reimbursed for out of pocket expenses, inconvenience and time involved by the provision of prepaid gift cards to an Australian retail outlet. A $20 gift card will be provided at the baseline visit and weeks 2, 4 and 6 (total reimbursement $80). For those participants who consent to the blood test an extra $10 will be provided at the baseline visit and week 6 (total reimbursement $100). If the study is terminated by the Investigator prior to completion, or a participant withdraws or is withdrawn from the study before completion, a pro-rata payment will be made at the discretion of the Investigator. The clinical trial insurance will reimburse participants for costs of medical care that occur as a result of complications directly related to participation in this study.

### Data management and monitoring

A screening log will be utilised to track potential participants and also record the counts of individuals approached, consented, meeting inclusion/exclusion criteria, withdrawals, and completion (in keeping with standard CONSORT diagram requirements). The case report form (CRF) will comprise of the hard copy questionnaires, clinical assessments and measures. These de-identified data will be retained in a secure room, in a locked filing cabinet, at each site. De-identified data from the CRFs will be entered into REDCap, which is a secure (encrypted to health service standard, housed on a server behind the University of Queensland firewall), web-based application for building and managing online surveys and databases. Delegated research assistants will be trained in, and responsible for, entering data into the database. Upon completion and resolution of monitoring and data management queries, the clinical trial database will be closed. A copy of the PICF will be stored in a secure room in a locked filing cabinet separate from the CRFs.

The Investigator, Project Manager or their delegate at each site will organise the retention of documentation relating to the study (source documents, informed consent forms, approvals) for a period of at least 15 years or the maximum time frame as determined by local regulations, whichever is the longest.

### Statistical analysis

We will compare demographic and clinical differences between the groups at baseline (Fisher exact test for nominal variables and Mann-Whitney test or independent sample t test for continuous variables). All data will be analysed, regardless of whether participants withdrew spontaneously or were withdrawn by the investigator. Efficacy will be assessed according to standard Intention to Treat (ITT) analytic procedures (i.e. for those who do not complete the 6-week study period, we will carry forward their last observation on the study outcomes). A secondary analysis will also be conducted including participants who (1) were in the trial for all 6 weeks, (2) were treatment adherent ≥80% and (3) had no other major protocol violations. Missing values will be addressed using a mixed-effects model repeated measures analysis which is robust in maintaining statistical properties of tests. Mean changes in clinical assessment will be assessed using mixed-model repeated-measure (MMRM) methods with treatment, week, and treatment-week interaction as fixed effects and intercept as the only random effect; baseline value will be the covariant. *P* values will be based on 2-tailed tests with significance levels of 0.05 corrected for multiple comparisons. A more detailed statistical analysis plan will be finalised before database lock.

### Monitoring and quality assurance

An independent Study Monitor will conduct study documentation reviews to monitor key features of the study prior to commencement, during and after study completion. These site visits will enable the Monitor to maintain current, personal knowledge of the study through review of the CRFs, comparison of CRF entries against the electronic data base (REDCap) and discuss the conduct of the study with the Investigator. The Monitor will be responsible for monitoring adherence to the approved study protocol, regulatory compliance including GCP and completion of the CRF.

Data quality will be ensured by performing data entry checks for consistency between the CRF and the data entry into REDCap database. These checks will be performed during data entry so that discrepancies can be resolved immediately. A data manager will later perform additional checks for completeness and plausibility of data. Resultant queries will be raised and resolved electronically by the data manager and the study centre.

### Data Safety Monitoring Board

An independent DSMB will be established specifically to monitor safety data and study trends throughout the duration of the trial to determine if the continuation of the trial is appropriate scientifically and ethically. The members of the DSMB serve in an independent capacity and will provide their expertise and recommendations to guide the clinical trial where required. The DSMB formally evaluates the protocol prior to the trial commencing and then is scheduled to assess trial progress (including review of all AEs) after the first 25 participants are recruited and at the end of the study with the completion of 52 participants. All serious AEs which are considered probably or definitely related to the trial product are immediately reported to the DSMB.

### Protocol amendments

Any amendments to the protocol will be submitted to the appointed HREC by the Chief Investigator for approval. Any approved amendments by the appointed HREC will be forwarded by the Chief Investigator for submission to each Research Governance Office.

No changes (amendments) to the Protocol will be implemented without prior approval from the Reviewing Ethics Committee.

### Dissemination of findings

Results will be disseminated in peer-reviewed publications and published in international journals. Authorship will be based upon the ICMJE guidelines. We do not intend to utilise professional writers. Our findings will also be summarised in several brochures, including one designed for feedback to participants in the study. Only group data will be reported. De-identified data may be made available upon request to the lead principal investigator. All adverse events will be reported in publications.

## Discussion

Currently available antipsychotics for the management of treatment-refractory schizophrenia have limited efficacy and a high burden of adverse effects [6]. Clozapine remains the most effective agent in those with schizophrenia who do not respond to antipsychotics [10] although its use is limited by its poor tolerability. Many novel therapeutics for schizophrenia currently under investigation have been unsuccessful in clinical trials [40]. Adjunctive sodium benzoate has now been shown to be effective in three clinical trials of people with treatment-refractory schizophrenia [22, 23, 25], although it was no more effective than placebo in people who were experiencing early illness [24]. In all studies, it had no more adverse effects than placebo. Taken together, there is a compelling case to further investigate sodium benzoate for the management of schizophrenia.

The described study will provide essential foundational information on the optimal dose and mechanism of action. Sodium benzoate, if effective, would be an important addition to the pharmacotherapies for treatment-resistant schizophrenia. It has the advantage over many other compounds on account of its affordability and favourable safety profile. Determination of the optimal dose and mechanism of action may pave the way for clinical trials which will determine if it is a treatment option for schizophrenia, an illness that continues to devastate the lives of so many individuals around the world.

## Data Availability

The datasets used and/or analysed during the study are available from the corresponding author on reasonable request.
